# Impact of HIV-1 Resistance-Associated Mutations on Susceptibility to Doravirine: Analysis of Real-World Clinical Isolates

**DOI:** 10.1128/AAC.01216-21

**Published:** 2021-11-17

**Authors:** Ernest Asante-Appiah, Johnny Lai, Hong Wan, Dongmei Yang, Elizabeth Anne Martin, Peter Sklar, Daria Hazuda, Christos J. Petropoulos, Charles Walworth, Jay A. Grobler

**Affiliations:** a Merck & Co., Inc., Kenilworth, New Jersey, USA; b Monogram Biosciences, South San Francisco, California, USA

**Keywords:** NNRTI, antiretroviral resistance, clinical isolates, doravirine

## Abstract

Clinical management of human immunodeficiency virus type-1 (HIV-1) infection may be negatively impacted by either acquired or transmitted drug resistance. Here, we aim to extend our understanding of the impact of resistance-associated mutations (RAMs) on the susceptibility of clinical isolates to the nonnucleoside reverse transcriptase inhibitor (NNRTI) doravirine. Clinical isolates from people living with HIV-1 undergoing routine testing for susceptibility to doravirine and other approved NNRTIs (etravirine, rilpivirine, efavirenz, and nevirapine) were collected from August 2018 to August 2019. Susceptibility in the presence/absence of NNRTI and nucleos(t)ide reverse transcriptase inhibitor (NRTI) mutations was determined using cutoffs for relative fold change in inhibition (ratio of the 50% inhibitory concentration [IC_50_] of patient virus compared with the IC_50_ of a wild-type reference strain). Biological cutoffs of 3- to 15-fold change were investigated for doravirine, with preestablished cutoffs used for the other NNRTIs. Of 4,070 clinical isolates, 42.9% had ≥1 NNRTI RAM. More isolates were susceptible to doravirine (92.5–96.7%) than to etravirine (91.5%), rilpivirine (89.5%), efavirenz (81.5%), or nevirapine (77.5%). Based on a 3-fold cutoff, doravirine susceptibility was retained in 44.7–65.8% of isolates resistant to another NNRTI and 28.5% of isolates resistant to all other tested NNRTIs. The presence of NRTI RAMs, including thymidine analog mutations, was associated with doravirine hypersusceptibility in some isolates, particularly in the absence of NNRTI RAMs. These results support the favorable resistance profile of doravirine and are of particular importance given the challenge posed by both acquired and transmitted resistance.

## INTRODUCTION

Since the 1987 approval of the first nucleoside reverse transcriptase inhibitor (NRTI) by the United States Food and Drug Administration, antiretroviral therapy (ART) has revolutionized treatment for people living with human immunodeficiency virus-1 (HIV-1) (1). Effective treatment for people living with HIV-1 (PLWH) both improves their health and life expectancy and eliminates the risk of onward viral transmission (2–4). Unfortunately, the clinical and immunologic benefits associated with ART are compromised by the emergence of drug-resistant viruses facilitated by inadequate viral suppression and high mutation rates (5, 6).

Transmitted drug resistance (TDR), i.e., resistance transmitted at the time of infection, reduces the number of first-line therapy options available to PLWH and potentially impacts their subsequent treatment response (6–8). One study estimated that, from 2003 to 2016, 13.9% of ART-naive PLWH had evidence of TDR (9). Within this population from the United States (US), TDR was more commonly attributable to nonnucleoside reverse transcriptase inhibitor (NNRTI) resistance-associated mutations (RAMs) (9). A more recent study conducted in PLWH in the US from 2014 to 2018 estimated that the prevalence of NNRTI mutations was 12% (10). Studies have documented a progressive increase in the prevalence of TDR across several geographic regions (9–12). Moreover, changing practices in ART may contribute additional mutations to TDR, particularly for NNRTIs (13).

Doravirine, a next-generation NNRTI with activity against HIV-1 viruses bearing common NNRTI RAMs, is approved in combination with other antiretroviral agents for the treatment of HIV-1 infection in adults with no antiretroviral treatment history (14–20). The approval of doravirine was based on analyses of 48-week data from two randomized, multicenter, double-blind, active-controlled phase 3 trials (DRIVE-FORWARD [NCT02275780] and DRIVE-AHEAD [NCT02403674]) in ART-naive PLWH (21, 22). Doravirine (in combination with two NRTIs) displayed noninferior efficacy compared with efavirenz or ritonavir-boosted darunavir (also with two NRTIs) at week 48. Doravirine was generally well tolerated over the course of both trials, with a favorable safety and lipid profile compared with darunavir/ritonavir, and significantly fewer neuropsychiatric events than efavirenz (21, 22). These findings were further supported by the 96-week results from the DRIVE-FORWARD and DRIVE-AHEAD trials, which demonstrated the extended efficacy and safety of doravirine (23, 24). Moreover, a phase 3, randomized, open-label, active-controlled, noninferiority trial (DRIVE-SHIFT [NCT02397096]) has shown doravirine to be a generally well-tolerated treatment option that can maintain viral suppression among those considering a switch in therapy (25–27).

Doravirine has demonstrated *in vitro* activity against both wild-type viruses and those bearing mutations typically associated with resistance to other NNRTIs. It displayed potent inhibition of HIV-1 replication against the most prevalent NNRTI-resistant variants, including K103N, Y181C, G190A, and K103N+Y181C (15, 16), and its favorable resistance profile is unique among NNRTIs (15, 16, 28). A small phase 2, multicenter, open-label, single-arm trial (DRIVE-BEYOND [NCT02629822]) supported *in vitro* findings that doravirine is active against HIV-1 with K103N and G190A mutations, with antiretroviral efficacy observed in individuals with a single baseline NNRTI RAM of K103N or G190A (29). Furthermore, outcomes from the phase 3 DRIVE-SHIFT noninferiority trial provided additional support for doravirine activity against NNRTI RAMs (25). At study entry, 24 participants had virus with NNRTI resistance mutations K103N, Y181C, and/or G190A; of the 23 who switched to DOR/3TC/TDF, 21 maintained viral suppression through 48 weeks. Of the two participants who discontinued early, both had maintained viral suppression as of their last study visit (25).

To further understand the real-world activity of doravirine against viruses containing RAMs, for both NNRTIs and NRTIs, we evaluated its phenotypic susceptibility in comparison with other NNRTIs, in a large panel of clinical isolates submitted for routine drug-resistance testing.

## RESULTS

### Clinical isolates and prevalence of NNRTI RAMs.

From August 2018 to August 2019, a total of 4,070 clinical isolates from PLWH were submitted to Monogram Biosciences (South San Francisco, CA, USA) for routine susceptibility testing and included in this analysis. Overall, 42.9% (*n* = 1,746) of the sample set had at least one NNRTI RAM and 23.4% (*n* = 953) of isolates had only one NNRTI RAM. The proportion of isolates with two NNRTI RAMs was 9.8% (*n* = 398), the proportion with a combination of three or more NNRTI RAMs was comparable to this at 9.7% (*n* = 395). As shown in Table 1, the most commonly identified NNRTI RAMs among the clinical isolates, irrespective of the presence of other NNRTI RAMs, were K103N (detected in 14.3% of isolates), V106I (5.4%), and Y181C (5.3%).

**TABLE 1:** Prevalence of clinical isolates bearing a common NNRTI RAM and respective doravirine susceptibility

Common NNRTI RAM^*a*^	Prevalence, n (%)^*b*^ (N = 4,070)	Doravirine fold-change, median (IQR)^*c*^
K103N	580 (14.3)	1.3 (0.8–2.4)
V106I	218 (5.4)	1.3 (0.8–2.7)
Y181C	214 (5.3)	2.2 (1.2–4.6)
V108I	126 (3.1)	2.2 (1.3–5.5)
K101E	123 (3.0)	1.5 (1.0–2.3)
G190A	99 (2.4)	1.8 (1.0–4.1)
E138K	56 (1.4)	1.6 (0.9–2.4)
K103N+Y181C	53 (1.3)	3.1 (1.2–5.1)

a NNRTI RAMs present in over 50 isolates were classed as common.

b Prevalence of isolates with RAMs (n) irrespective of presence of any other NNRTI RAMs in total of clinical isolates.

c Relative to wild type.

IQR, interquartile range; NNRTI, nonnucleoside reverse transcriptase inhibitor; RAM, resistance-associated mutation.

### Susceptibility of clinical isolates to NNRTIs.

The percentages of clinical isolates susceptible to etravirine, rilpivirine, efavirenz, and nevirapine were 91.5%, 89.5%, 81.5%, and 77.5%, respectively. At a 3-fold biological cutoff, the percentage of isolates susceptible to doravirine was 92.5%; use of 5-, 10-, and 15-fold cutoff values increased the proportion of susceptible isolates to 94.5%, 96.0%, and 96.7%, respectively. Clinical isolates that bore common NNRTI RAMs (detected alone or in combination with other NNRTI substitutions) were found to be susceptible to doravirine; almost all median fold change values were below a 3-fold biological cutoff (Table 1).

**FIG 1:**
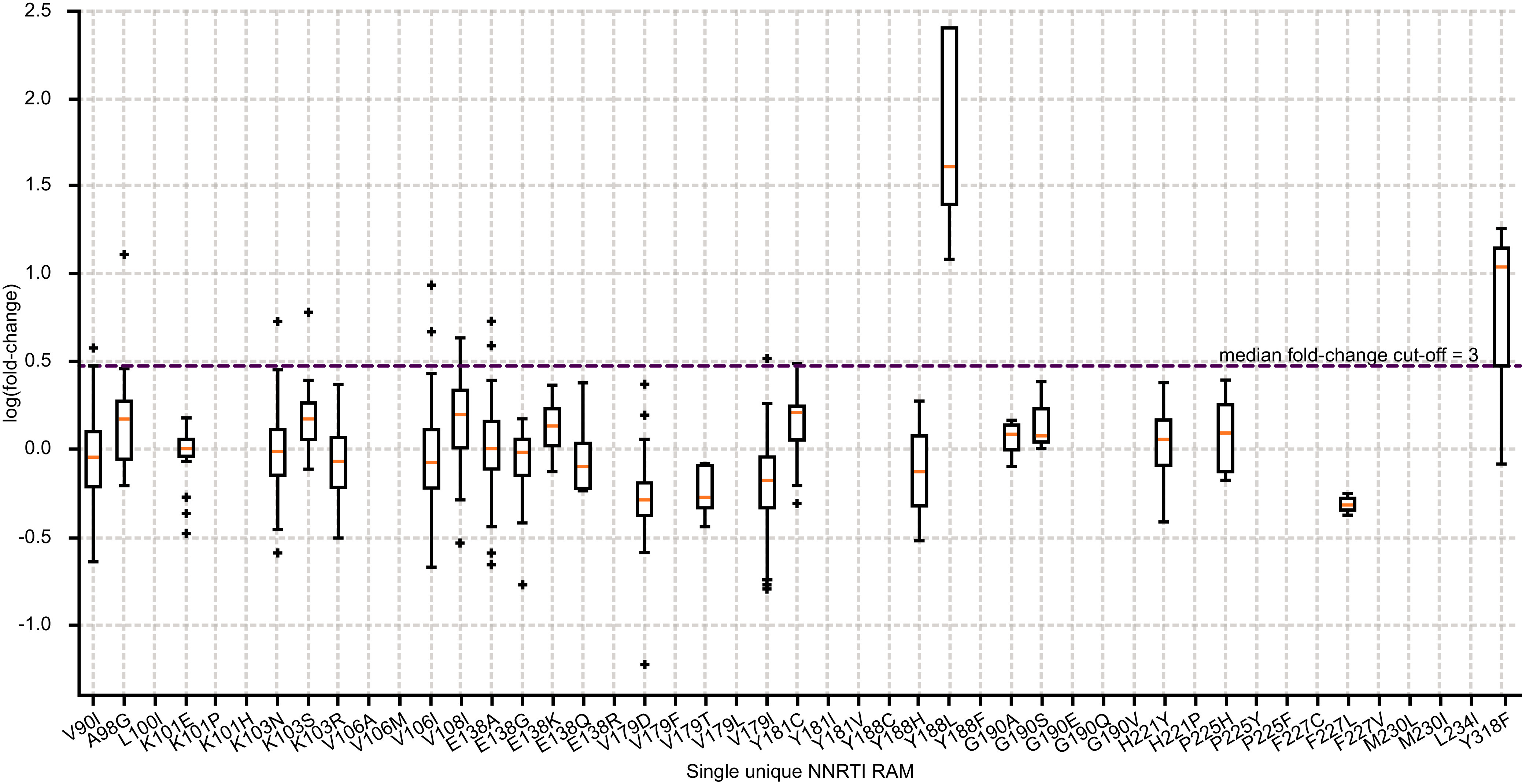
Doravirine susceptibility among clinical isolates bearing a single unique NNRTI RAM; red line represents the median fold change cutoff 3. NNRTI, nonnucleoside reverse transcriptase; RAM, resistance-associated mutation.

Most clinical isolates with a single unique NNRTI RAM were susceptible to doravirine. Only samples with Y188L or Y318F NNRTI substitutions displayed a median fold change above the 3-fold biological cutoff (Table 2; Fig. 1). The inhibitory quotient (IQ), defined as the ratio of the clinical trough concentration to the IC_50_ concentration, was also calculated for each clinical isolate with a single unique NNRTI RAM, and was found to be >40 for all isolates, except those with Y188L or Y318F substitutions (Fig. S1). In the evaluation of the relationship between the number of NNRTI RAMs and the susceptibility of clinical isolates to NNRTIs, the median doravirine fold change ranged from 0.9 to 3.0 for isolates with one to four NNRTI RAMs (approximately 96.6% of samples with RAMs). However, isolates that had a combination of five or more NNRTI RAMs had a median fold change of 5.6, indicating reduced susceptibility to doravirine (Table 3). In comparison, only isolates with one NNRTI RAM (approximately 54.6% of samples with RAMs) had median fold change values below the established cutoffs for efavirenz (3.0) and nevirapine (4.5), and only isolates with one or two NNRTI RAMs (approximately 77.4% of samples with RAMs) had median fold change values below the established cutoffs for etravirine (2.9) and rilpivirine (2.0), suggesting a reduction in their susceptibility to these NNRTIs (Table 4).

**TABLE 2:** Prevalence of clinical isolates bearing a single unique NNRTI RAM and respective doravirine susceptibility

Single unique NNRTI RAM	Prevalence, n (%) (N = 4,070)	Doravirine fold-change, median (IQR)^*a*^
K103N	237 (5.8)	1.0 (0.7–1.3)
V90I	104 (2.6)	0.9 (0.6–1.3)
V106I	102 (2.5)	0.8 (0.6–1.3)
E138A	61 (1.5)	1.0 (0.8–1.4)
K103R	49 (1.2)	0.9 (0.6–1.2)
V179D	45 (1.1)	0.5 (0.4–0.6)
V108I	26 (0.6)	1.6 (1.0–2.2)
Y181C	21 (0.5)	1.6 (1.2–1.8)
K101E	17 (0.4)	1.0 (0.9–1.1)
A98G	16 (0.4)	1.5 (0.9–1.9)
Y188L	15 (0.4)	41.0 (25.0–250.0)
P225H	11 (0.3)	1.2 (0.7–1.8)
K103S	10 (0.3)	1.5 (1.1–1.8)
E138G	9 (0.2)	1.0 (0.7–1.1)
E138K	7 (0.2)	1.4 (1.1–1.7)
G190A	6 (0.2)	1.2 (1.0–1.4)
E138Q	5 (0.1)	0.8 (0.6–1.1)
H221Y	4 (0.1)	1.2 (0.8–1.5)
Y318F	3 (<0.1)	11.0 (3.0–14.1)
Y188H	2 (<0.1)	0.8 (0.5–1.2)

a Relative to wild type.

IQR, interquartile range; NNRTI, nonnucleoside reverse transcriptase inhibitor; RAM, resistance-associated mutation.

**TABLE 3:** Prevalence of clinical isolates according to the number of NNRTI RAMs and respective susceptibility to doravirine

No. of RAMs	Prevalence, n (%) (N = 4,070)	Doravirine fold-change, median (IQR)	*P*-value^*a*^
1	953 (23.4)	0.9 (0.6–1.3)	<0.0001
2	398 (9.8)	1.3 (0.8–2.4)	<0.0001
3	219 (5.4)	2.3 (1.2–7.7)	<0.0001
4	108 (2.7)	3.0 (1.4–13.2)	<0.0001
≥5	68 (1.7)	5.6 (2.4–33.6)	<0.0001

a *P-*value from the Mann-Whitney U to test whether fold-change from a randomly selected group of X number of RAMs is significantly greater or less than the fold-change from a randomly selected group without any RAMs (wild-type).

IQR, interquartile range; NNRTI, nonnucleoside reverse transcriptase inhibitor; RAM, resistance-associated mutation.

**TABLE 4:** Susceptibility of clinical isolates to nondoravirine NNRTIs according to the number of NNRTI RAMs

No. of RAMs	Fold-change, median (IQR)			
	Efavirenz	Etravirine	Nevirapine	Rilpivirine
1	1.1 (0.7–2.9)	0.8 (0.6–1.1)	1.3 (0.6–12.0)	0.8 (0.6–1.1)
2	3.7 (1.2–16.0)	1.1 (0.7–2.2)	26.0 (1.9–250.0)	1.1 (0.7–2.0)
3	15.0 (2.5–150.0)	3.1 (1.2–13.0)	250.0 (40.5–250.0)	3.6 (1.3–17.5)
4	27.0 (5.0–150.0)	4.6 (1.6–21.7)	250.0 (145.6–250.0)	5.4 (1.9–43.9)
≥5	150.0 (12.0–150.0)	27.5 (7.2–97.0)	250.0 (250.0)	38.4 (5.9–150.0)

Fold-change cutoffs established by Monogram Biosciences: efavirenz: 3.0, etravirine: 2.9, nevirapine: 4.5, rilpivirine: 2.0.

IQR, interquartile range; NNRTI, nonnucleoside reverse transcriptase inhibitor; RAM, resistance-associated mutation.

### Cross-resistance of NNRTI-resistant clinical isolates.

Doravirine exhibited the broadest susceptibility profile of all the tested NNRTIs with an estimated 44.7–65.8% of samples resistant to another NNRTI remaining susceptible to doravirine (Table 5). In contrast, nevirapine displayed the most limited susceptibility profile with activity against only 1.6–9.3% of samples that were resistant to another NNRTI. Of all clinical isolates assessed, 5.6% (*n* = 228) exhibited resistance to all the other NNRTIs; of these, 28.5% (*n* = 65) retained susceptibility to doravirine.

**TABLE 5:** Proportion of NNRTI-resistant clinical isolates that are susceptible to other NNRTIs

NNRTI	Percentage of resistant samples susceptible to other NNRTIs, %				
	Doravirine-resistant^*a*^	Efavirenz-resistant	Etravirine-resistant	Nevirapine-resistant	Rilpivirine-resistant
Doravirine-susceptible^*a*^		62.2	44.7	65.8	45.0
Efavirenz-susceptible	17.1		31.0	18.7	28.1
Etravirine-susceptible	45.2	68.8		65.0	24.4
Nevirapine-susceptible	9.3	1.6	6.0		8.7
Rilpivirine-susceptible	31.9	59.4	5.6	57.4	

a A doravirine cutoff 3-fold was used for this comparison.

NNRTI, nonnucleoside reverse transcriptase inhibitor.

### Impact of NRTI mutations on the susceptibility of clinical isolates to doravirine.

The most prevalent mutation associated with NRTI resistance was M184I/V, detected in 17.7% or 6.4% of isolates in the presence or absence of any NNRTI RAMs, respectively. The presence of the common NRTI RAMs included in this study (K65R, L74I/V and M184I/V) did not confer resistance to doravirine in the absence of NNRTI RAMs; all of the isolates that bore these mutations were found to be susceptible to doravirine. However, the extent of susceptibility to doravirine in isolates with K65R, L74I/V and/or M184I/V NRTI RAMs was found to vary depending on the presence or absence of NNRTI RAMs. Isolates bearing both NRTI and NNRTI RAMs were associated with median fold changes between 0.8 and 2.0, while those with RAMs only for NRTIs were associated with median fold changes ranging from 0.5 to 0.6. The proportions of clinical isolates with NRTI RAMs that showed hypersusceptibility to doravirine (median fold change <0.4) were greater in the absence of NNRTI RAMs than in their presence. Compared with other NRTI RAMs, clinical isolates that bore the K65R NRTI RAM (with or without M184I/V) were associated with lower median fold changes, and higher proportions of them showed hypersusceptibility, both in the absence and presence of NNRTI RAMs (Table 6). The presence of thymidine analog mutations (TAMs) had an effect similar to that observed with the non-TAM NRTI RAMs; all isolates with these mutations were found to be doravirine susceptible and doravirine hypersusceptibility was more prevalent in the absence rather than in the presence of NNRTI RAMs. However, the proportion of clinical isolates with hypersusceptibility to doravirine was higher in those bearing the M184I/V NRTI RAM than those containing NRTI TAMs (Table 6).

**TABLE 6:** Clinical isolates with NRTI RAMs or NRTI TAMs with/without M184I/V and respective doravirine susceptibility in the absence and presence of NNRTI RAMs

	Samples without NNRTI RAMs (N = 2,323)			All samples (+/-NNRTI RAMs) (N = 4,070)		
Mutation	Prevalence, n (%)	Fold-change, median (IQR)	Hypersusceptibility^*a*^, %	Prevalence, n (%)	Fold-change, median (IQR)	Hypersusceptibility^*a*^, %
NRTI RAMs						
K65R	38 (1.6)	0.5 (0.4–0.7)	31.6	115 (2.8)	0.8 (0.5–2.1)	17.4
L74I/V	6 (0.3)	0.5 (0.5–0.6)	16.7	80 (2.0)	2.0 (0.8–5.5)	3.8
M184I/V	260 (11.2)	0.6 (0.4–0.9)	18.9	721 (17.7)	0.9 (0.5–2.0)	11.4
M184I/V + K65R	35 (1.5)	0.5 (0.4–0.7)	31.4	105 (2.6)	0.8 (0.5–2.0)	18.1
M184I/V + L74I/V	5 (0.2)	0.6 (0.5–0.6)	20.0	71 (1.7)	1.8 (0.8–5.4)	4.2
NRTI TAMs with/without M184I/V						
TAMs	73 (3.1)	0.7 (0.6–1.0)	11.0	227 (5.6)	1.1 (0.7–2.4)	5.3
M184I/V	199 (8.6)	0.6 (0.4–0.9)	19.6	479 (11.8)	0.8 (0.5–1.5)	13.2
TAMs + M184I/V	61 (2.6)	0.6 (0.4–0.7)	16.4	242 (5.9)	1.2 (0.6–5.4)	7.9

a Fold-change <0.4; *P* value <0.001; the *P* value from the Mann-Whitney U to test whether the fold-change differs between samples with or without NRTI RAMs.

IQR, interquartile range; NNRTI, nonnucleoside reverse transcriptase inhibitor; NRTI, nucleoside reverse transcriptase inhibitor; RAM, resistance-associated mutation; TAM, thymidine analog mutations.

## DISCUSSION

This analysis of real-world clinical isolates obtained from treatment-naive and treatment-experienced PLWH undergoing routine resistance testing supports previous observations that doravirine is active against viruses bearing NNRTI RAMs (15, 16, 29). In comparison with the other NNRTIs tested (etravirine, rilpivirine, efavirenz, and nevirapine), a higher percentage of isolates showed susceptibility to doravirine. This activity was maintained in the presence of almost all the NNRTI RAMs that were tested against doravirine, including common mutations such as K103N, V106I, and Y181C. In addition, doravirine IQs were >40 for the vast majority of the isolates with single unique NNRTI RAMs. Doravirine has previously been shown to have higher IQs compared with rilpivirine or efavirenz for a range of NNRTI-associated RAMs, including K103N and G190A, which had IQs of 68 and 56, respectively, in the current study (16). K103N, the NNRTI RAM most commonly detected within clinical isolates in our analysis, is a mutation previously shown to be associated with high-level resistance to efavirenz and nevirapine, conferring cross-resistance between the two drugs (30, 31). Though resistance to doravirine was observed in some isolates with only a single unique RAM present (Y188L or Y318F), generally, a minimum of five NNRTI RAMs was required to reduce susceptibility to doravirine based on the 3-fold biological cutoff. In contrast, the presence of only two or three NNRTI RAMs was sufficient to reduce the susceptibility of clinical isolates to the other NNRTIs tested (etravirine, rilpivirine, efavirenz, and nevirapine) based on the established clinical cutoffs. These findings are consistent with two recent reports on the prevalence of doravirine resistance-associated substitutions in European PLWH (32, 33). The prevalence of doravirine RAMs was lower than those associated with other NNRTIs in treatment-naive and treatment-experienced patients (32, 33).

Among clinical isolates that were resistant to at least one other NNRTI, doravirine demonstrated the broadest susceptibility profile; in the majority of instances, susceptibility of these isolates to another NNRTI was highest with doravirine compared with the other NNRTIs evaluated. More importantly, doravirine retained this activity in over a quarter of the isolates that were resistant to all the other NNRTIs tested. A separate *in vitro* study of HIV-1 viruses with intermediate-to-high-level resistance to rilpivirine, etravirine, efavirenz and nevirapine found that doravirine susceptibility decreased with increasing numbers (≥3) of NNRTI RAMs (34). Five of the 10 recombinant clones investigated had comparable or greater suspectibility than other NNRTIs, and it was concluded that as the presence of multiple mutations that confer resistence to older NNRTIs can also result in cross-resistence to doravirine in some cases, the additional use of genotypic interpretation algorithms may be beneficial in informing treatment decisions for heavily treatment-experienced individuals (34)

Our results also suggest that viruses bearing certain NRTI RAMs impact doravirine susceptibility. The presence of common NRTI RAMs (such as K65R and/or M184I/V) was associated with doravirine hypersusceptibility in some clinical isolates, upwards of 11.4%, especially in the absence of NNRTI RAMs. The highest proportion of isolates to demonstrate hypersusceptibility was 31.6%, recorded in isolates with the K65R RAM (and no NNRTI RAMs).

The findings we report here that multiple mutations are generally necessary for the development of doravirine resistance could explain the low rate of acquired doravirine resistance that was observed in clinical trial participants (35). In a recently published review, analysis of treatment-naive participants in four phase 2 and 3 clinical trials of doravirine (P007 [NCT01632345], DRIVE-BEYOND, DRIVE-FORWARD, and DRIVE-AHEAD) revealed that less than 1% of participants experienced treatment failure with doravirine resistance (35). The generation of the appropriate data set needed to establish clinical cutoffs cannot always be obtained due to both the design and the results of clinical trials. Here, the limited number of treatment failures in the pivotal phase 3 treament-naive studies (DRIVE-FORWARD and DRIVE-AHEAD) has contributed to the inability to establish a clinical cutoff for DOR. In total, only 8 participants were detected with DOR phenotypic resistance over a duration of 96 weeks (23, 35). In addition, among those participants with viruses exhibiting phenotypic resistance to DOR, the combination of mutations all conferred >100-fold loss in potency, precluding the ability to determine a breakpoint at which virologic response begins to decline, which is needed to establish a lower clinical cutoff. The sensitivity analysis using 3-, 5-, 10- and 15-fold biological cutoffs suggest a majority of clinical isolates (>92%) would be susceptible to DOR. These findings are particularly relevant in certain geographic regions or in clinical situations where TDR is of concern.

TDR has been recorded in almost all regions where drug-resistance testing has been conducted (12). With the TDR incidence on the rise across several geographic regions, resistance testing in ART-naive individuals is recommended at the time of diagnosis to detect potential RAMs (9, 11, 12, 36).

One limitation of this study is that clinical details such as the treatment group and the reason for sample submission are unknown. Thus, there could be no assessment of clinical outcomes based on the resistance data. While this analysis provides valuable information on the susceptibility of clinical isolates to doravirine, these observations will need to be confirmed in clinical studies to determine the efficacy of DOR for PLWH with NNRTI resistance. Furthermore, the prevalence of HIV subtypes among the clinical isolates is unknown but expected to be subtype B, seeing as the study was US-based (37); hence the impact of NNRTI mutations on DOR susceptibility in other HIV subtypes remains to be elucidated.

In summary, our results support previous findings that doravirine possesses a favorable resistance profile that is unique among NNRTIs. This is an important consideration given the growing challenge posed by both acquired and transmitted drug resistance.

## MATERIALS AND METHODS

### Impact of NNRTI RAMs on the susceptibility of clinical isolates to NNRTIs.

NNRTI genotypic and phenotypic susceptibility data were obtained using the PhenoSense GT and PhenoSense GT Plus Integrase HIV drug-resistance assays as described previously (Monogram Biosciences, South San Francisco, CA, USA) (38). Samples were collected from clinical isolates of treatment-naive and treatment-experienced PLWH undergoing routine testing for susceptibility to doravirine, and four other approved NNRTIs (etravirine, rilpivirine, efavirenz, and nevirapine), at Monogram Biosciences (South San Francisco, CA, USA) over a 1-year period (August 2018 to August 2019). The prevalence of NNRTI RAMs in these clinical isolates was determined by monitoring the following substitutions in reverse transcriptase: V90I, A98G, L100I, K101E/H/P, K103N/R/S, V106A/I/M, V108I, E138A/G/K/Q/R, V179D/F/I/L/T, Y181C/I/V, Y188C/F/H/L, G190A/E/Q/S/V, H221P/Y, P225F/H/Y, F227C/L/V, M230I/L, L234I, Y318F. Phenotypic susceptibility of the clinical isolates is expressed as the fold change, which is the ratio of the 50% inhibitory concentration [IC_50_] of patient virus compared with the IC_50_ of a wild-type reference strain. Doravirine IQs were calculated as the ratio of the clinical trough concentration to the IC_50_, with a clinical trough concentration of 830 nM and a wild-type IC_50_ of 12 nM (16).

The susceptibility of isolates to NNRTIs was determined with the use of fold change cutoffs established by Monogram Biosciences: etravirine, 2.9; rilpivirine, 2.0; efavirenz, 3.0; nevirapine, 4.5. As the absence of an appropriate clinical data set means there is currently no established clinical cutoff for doravirine, biological cutoffs of 3-, 5-, 10-, or 15-fold change were used.

Doravirine susceptibility among the clinical isolates was also assessed following stratification by the presence of highly common NNTRI RAMs. Additionally, the susceptibility of clinical isolates to NNRTIs was evaluated after stratification by the total number of NNRTI RAMs present. The extent of cross-resistance among samples was similarly evaluated; first by determining the percentage resistant to a particular NNRTI, then the percentage of that subset susceptible to each of the other tested NNRTIs.

### Impact of NRTI mutations on the susceptibility of clinical isolates to doravirine.

The prevalence of NRTI mutations in the tested clinical isolates was determined by monitoring the NRTI RAMs K65R, L74I/V, and/or M184I/V; and the NRTI TAMs M41L, D67N, K70R, L210W, T215F/Y, and K219E/Q. The susceptibility of isolates to doravirine was assessed based on a biological cutoff 3-fold change; isolates with a fold change below 0.4 were categorized as displaying hypersusceptibility to doravirine. Doravirine susceptibility was also assessed following stratification by the presence and absence of NNTRI RAMs.

### Data availability.

Merck Sharp & Dohme Corp., a subsidiary of Merck & Co., Inc., Kenilworth, NJ, USA’s data sharing policy, including restrictions, is available at http://engagezone.msd.com/ds_documentation.php. Requests for access to the clinical study data can be submitted through the EngageZone site or via email to dataaccess@merck.com.
