# Supplementary material for: Impact of HIV-1 Resistance-Associated Mutations on Susceptibility to Doravirine: Analysis of Real-World Clinical Isolates
Source: Antimicrob Agents Chemother. 2021 Nov 17;65(12):e01216-21. doi: 10.1128/AAC.01216-21 (PMC8597775; doi:10.1128/AAC.01216-21)
Supplement: Supplemental file 1 — Supplemental material. Download aac.01216-21-s0001.pdf, PDF file, 0.2 MB [file aac.01216-21-s0001.pdf]

Supplementary Information

Impact of HIV-1 Resistance-associated Mutations on Susceptibility to Doravirine: Analysis of Real-world Clinical Isolates

Ernest Asante-Appiah,[a]# Johnny Lai,[b] Hong Wan,[a] Dongmei Yang,[b] Elizabeth Anne Martin,[a] Peter Sklar,[a] Daria Hazuda,[a] Christos J. Petropoulos,[b] Charles Walworth,[b] Jay A. Grobler[a]

[a]Merck & Co., Inc., Kenilworth, New Jersey, USA

[b]Monogram Biosciences, South San Francisco, California, USA

#Address correspondence to: Ernest Asante-Appiah, Ph.D,

ernest_asanteappiah@merck.com

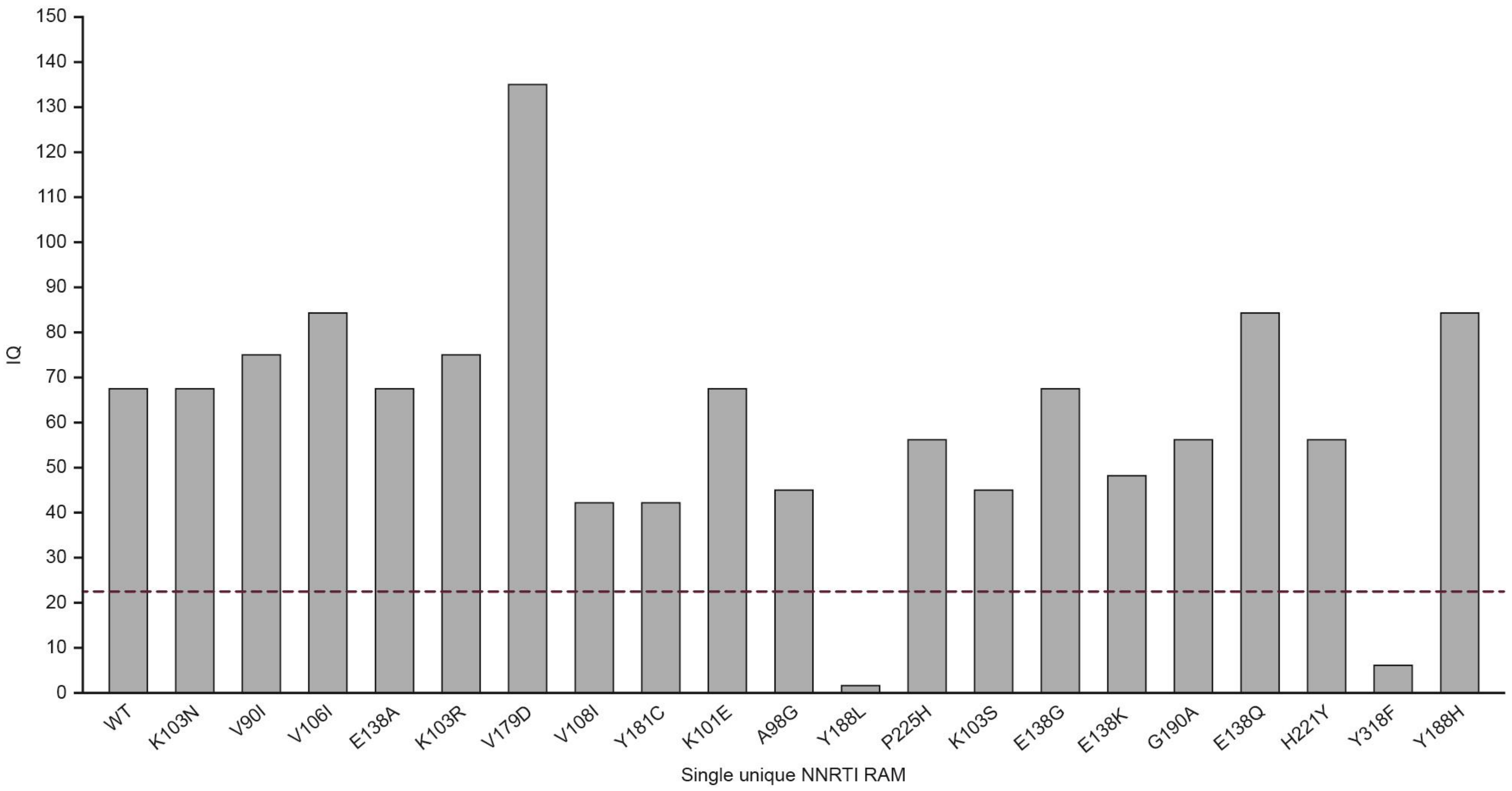

FIG S1 Inhibitory quotients of doravirine against clinical isolates bearing a single unique NNRTI RAM; dotted line represents the biological fold-change cut-off of 3.

IQ, inhibitory quotient; NNRTI, non-nucleoside reverse transcriptase; RAM, resistance-associated mutation.
